# Structural dynamics: review of time-resolved cryo-EM

**DOI:** 10.1107/S2059798322006155

**Published:** 2022-07-21

**Authors:** Märt-Erik Mäeots, Radoslav I. Enchev

**Affiliations:** a The Visual Biochemistry Laboratory, The Francis Crick Institute, 1 Midland Road, London NW1 1AT, United Kingdom

**Keywords:** cryo-EM, time-resolved cryo-EM, sample preparation, structural biology, structural dynamics

## Abstract

The structural determination of biological macromolecules has been transformative for understanding biochemical mechanisms and developing therapeutics. However, the ultimate goal of characterizing how structural dynamics underpin biochemical processes has been difficult. This is largely due to significant technical challenges that hinder data collection and analysis on the native timescales of macromolecular dynamics. Single-particle cryo-EM provides a powerful platform to approach this challenge, since samples can be frozen faster than the single-turnover timescales of most biochemical reactions. In order to enable time-resolved analysis, significant innovations in the handling and preparation of cryo-EM samples have been implemented, bringing us closer to the goal of the direct observation of protein dynamics in the milliseconds to seconds range. Here, the current state of time-resolved cryo-EM is reviewed and the most promising future research directions are discussed.

## Abstract 2

Time-resolved cryo-EM is an emerging technique in structural biology that allows the user to capture structural states which would otherwise be too transient for standard methods. There has been a resurgence in technical advancements in this field in the last five years and this review provides a summary of the technical highlights.

## 1. Introduction

The structural study of biological macromolecules is of great utility in understanding biological mechanism. Over the last decade, cryo-EM and single-particle analysis experienced the fastest growing adoption of any structural biology technique, and revolutionized the study of macromolecular complexes and membrane proteins, as well as clinically relevant ordered aggregates (Curry, 2015 ▸; Fitzpatrick *et al.*, 2017 ▸; Ognjenović *et al.*, 2019 ▸). More recently, the technique has been utilized for pharmaceutical research and development in industrial settings (Hu *et al.*, 2018 ▸; Renaud *et al.*, 2018 ▸).

The function of a biological macromolecule, as a rule, is mediated by structural transitions that enable and modulate its ability to bind other molecules and/or catalyse a chemical reaction. However, not all of these functionally relevant states are equally accessible to structural determination by current methods. Many proteins perform their function within milliseconds by sampling high-energy intermediate structural states that are of great relevance to function but have very low occupancy under equilibrium conditions (Henzler-Wildman & Kern, 2007 ▸). This poses a major practical challenge to traditional structural biology approaches, which largely rely on sample-preparation methods that are orders of magnitude slower than the native timescale of the studied biochemical process. Consequently, stabilized conformations that can be averaged during data processing are necessary for high-resolution reconstructions. This is often not possible for active conformations, such as those requiring ATP hydrolysis, catalysis or transient binding, as they are not stable for long time periods and are not routinely resolved. These conformations represent critical points in macromolecular function and are likely to harbour many additional insights into both the mechanism itself and potential therapeutic targets by revealing key secondary-structure transformations that could be targeted. This information will be key to increasing the size of the druggable proteome where current structural approaches have not yielded the desired insight. Even though it is widely accepted that these off-equilibrium structures would be insightful for further biological discovery, they remain severely understudied relative to their importance. This has motivated many attempts to develop new methods for structural biology which can capture these states.

All three main structural biology techniques, X-ray crystallography, NMR and cryo-EM, are in principle adaptable to time-resolved structural studies and each harbours particular advantages and challenges. X-ray crystallography is a well established, reliable and broadly used technique for determining high-resolution structures. The downside is that it requires the sample to be stabilized in a crystalline state, rendering it very difficult to study the full complement of native macromolecular dynamics. Thus, in order to initiate a biochemical reaction shortly before taking a diffraction-pattern data set, time-resolved X-ray crystallographic methods either focus on small reactants that can diffuse into the crystal lattice (Liu & Lee, 2019 ▸) or form the crystals in the presence of caged compounds that can be activated by a light pulse (Ellis-Davies, 2007 ▸). Time-resolved crystallographic techniques can cover a wide range in time, from femtoseconds to milliseconds; however, the choice of samples is more limited, as discussed by Brändén & Neutze (2021 ▸). A technological alternative is to use NMR to time-resolve biochemical reactions. This, while theoretically possible, becomes prohibitively expensive in terms of time and effort, so it is usually used to ask very specific structural questions rather than solve the entire time-resolved state itself (Gołowicz *et al.*, 2020 ▸). There are also limitations on the size of samples which can be studied by NMR, which is considered to be around 30 kDa at the high end.

A defining characteristic of NMR and X-ray crystallo­graphic data analysis, imposed by the nature of the studied sample, is that it operates on ensembles, averaging any underlying conformational dynamics. A notable exception is presented by single-particle X-ray free-electron laser (XFEL) methods, which are still to realize their full potential and are under active development. A discussion of the perspectives of this time-resolved technique can be found in Liu & Lee (2019 ▸). This contrasts with single-particle electron-microscopy approaches, which apply statistical analysis to individual molecular images, and averaging only occurs after unique groups of particles have been identified (Scheres, 2012 ▸). The general technique of cryo-electron microscopy (cryo-EM) and single-particle analysis (referred to merely as cryo-EM for the remainder of this review) has been rapidly developing through both software and hardware solutions, which have been reviewed elsewhere (Kühlbrandt, 2014 ▸; Cheng *et al.*, 2015 ▸; Nogales & Scheres, 2015 ▸; Passmore & Russo, 2016 ▸; Earl *et al.*, 2017 ▸). As such cryo-EM is being applied to more and more complex biological questions, with the major limitation to its applicability now stemming from the ability to purify the sample biochemically as well as its structural stability. Although intrinsically disordered proteins cannot be studied, most biological macromolecules exhibit a more limited, albeit significant, tendency to adopt multiple flexible conformations. Cryo-EM has several key advantages for studying such rare structural states. Cryo-EM sample preparation is compatible with ongoing biochemical reactions: the sample remains in solution and in a suitable buffer for protein activity until the point of vitrification. Additionally, the vitrification process itself is rapid and mainly depends on the physical speed of the grid entering the cryogen, which tends to take around 3 ms, while freezing occurs much faster at the point of contact (Kasas *et al.*, 2003 ▸). During vitrification, the sample also becomes frozen in time, meaning that any structural states that are present in solution can be studied. The cryo-EM field is already rapidly developing software solutions to mine data sets for populations of discrete or different structural states, including rare ones (Frank & Ourmazd, 2016 ▸; Punjani *et al.*, 2020 ▸; Wu *et al.*, 2020 ▸; Chen & Ludtke, 2021 ▸; Punjani & Fleet, 2021 ▸; Zhong *et al.*, 2021 ▸). These approaches use machine learning or deep learning to understand the population distribution of different states in a given data set and aspire to resolve the entire continuum of conformations of a macromolecule automatically.

Even though all accessible states are likely to be occupied under equilibrium conditions, and as a simplification most biochemical reactions are at equilibrium prior to being applied to the grid and frozen, many functionally relevant conformations can be of very low occupancy (Ourmazd, 2019 ▸). Thus, there are limitations to capturing these rare states in terms of instrument time and computational resources, due to there being a minimum number of representative images required to produce a structure. When the occupancy of the state is well below 1%, for example, one would require hundreds of times more data to produce the same resolution structure compared with the major classes. This category includes the majority of catalytical and transition states at equilibrium. If transient intermediates are to be solved, then being able to control biochemical reactions and incubation times at the same time as preparing cryo-EM samples is a very attractive alternative for enriching the sample with these states. This has led to the development of new methods that would allow precise time control over a biochemical reaction prior to freezing. If the timescale of the reaction of interest is known, it would be possible to freeze the sample at the point where the transient structure is most abundant. This method is known as time-resolved cryo-EM (trEM). To achieve trEM in the subsecond range, several major changes need to be made to the sample-preparation process. This involves rapidly mixing reactants to initiate a reaction and incubating the reaction for a desired time, followed by thinning the sample and freezing. This represents a great challenge for methods development, as large changes need to be made to existing protocols and tools. Progress on all these facets of the problem will be reviewed below, followed by an outlook on the future prospects of trEM.

## 2. Rapid reaction initiation

**Figure 1:**
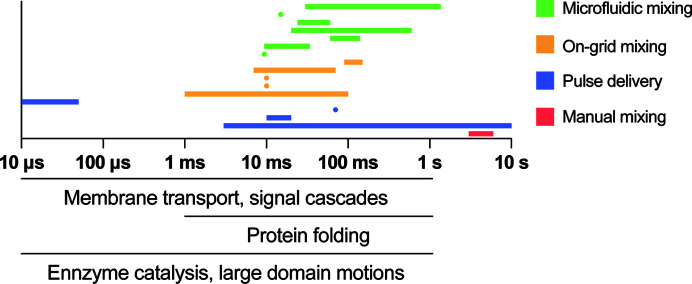
Overview of timescales achieved by time-resolved cryo-EM techniques and the categories of technologies that were used. Individual data points are listed in Table 1 ▸. Typical timescales of biological macromolecule motions are indicated (Henzler-Wildman & Kern, 2007 ▸).

**Figure 2:**
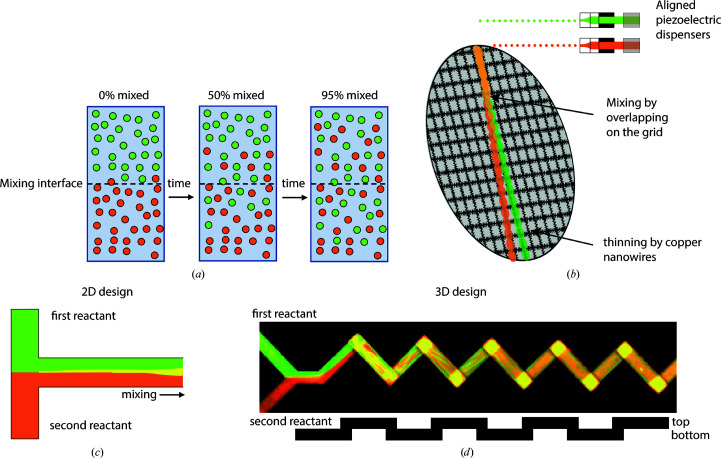
Sample-mixing overview. (*a*) Conceptual diagram representing ‘snapshots’ in time during diffusive mixing across a liquid interface. Because diffusion is the driving force in many mixing applications, it is critical to increase the surface-to-volume ratio to speed up mixing. (*b*) On-grid mixing of the type described in Jain *et al.* (2012 ▸), where two sequential jets of sample are applied on top of each other and mixing occurs at the overlap. The depiction here shows jets at slight angles for illustrative purposes; in the actual implementation the jets are aligned. (*c*) Parallel lamination mixing, where multiple fluid streams are connected into one, generating diffusive mixing at the interface. In the case of a T-mixer geometry there is a single mixing interface along the centre of the channel. (*d*) Passive microfluidic mixing, repeatedly inducing breakup and re-merging of the liquid layers, generates many thin layers (seen as striations), thus increasing the surface-to-volume ratio of the mixing interfaces.

The first necessary condition for monitoring the structural transitions that underpin a biochemical process is to rapidly and reliably initiate the biochemical reaction in a synchronous manner. In most biochemical assays this is performed by hand through vigorous pipetting. The speed and efficiency of mixing in these cases are mostly ignored, as the single-turnover kinetics of most studied processes are much faster than the timescales that can be reliably monitored in a manually executed assay. However, when more rapid assays are performed, mixing becomes an increasingly vital part of the process to the point of being rate-limiting if times below milliseconds are to be achieved (Fig. 1 ▸). Proteins diffuse in free solution with a rate within an order of magnitude of 100 µm^2^ s^−1^ (Nauman *et al.*, 2007 ▸), meaning that for any reactions that are less than a second in expected duration mixing by diffusion alone will not be fast enough for the reaction to initiate simultaneously. Mixing in most cases relies on the contact of two layers with different species being thin enough for diffusion to equilibrate them rapidly (Fig. 2 ▸ *a*).

The potential for coupling cryo-EM to time-resolved studies was pursued long before the ‘resolution revolution’. Early work on trEM mostly relied on manual mixing, either on the grid itself (Siegel *et al.*, 1989 ▸) or first in a test tube (Mandelkow *et al.*, 1991 ▸; Heymann *et al.*, 2004 ▸), followed by conventional application to the grid and blotting (Siegel *et al.*, 1989 ▸; Talmon *et al.*, 1990 ▸). Such approaches were applied to study microtubule dynamics, membrane fusion and phospholipid phase changes. Of necessity, the kinetics of the studied processes in this manner were slow enough to be outpaced by human operators. Despite this lack of general applicability, the method is still successfully applied today in special cases (Fischer *et al.*, 2010 ▸; Mulder *et al.*, 2010 ▸; Miller *et al.*, 2019 ▸). In a noteworthy example it reconciled the mechanism of origin loading by the MCM complex (Miller *et al.*, 2019 ▸). However, if reaction times under one second are to be studied, a much more rapid and reliable mixing technology is needed to facilitate the required time resolution (Fig. 1 ▸).

One approach that provides superior time resolution is applying the first reactant onto the sample support grid and blotting it into a thin film, and subsequently plunging it through an aerosol of small droplets of the second reactant (Fig. 2 ▸ *b*; Berriman & Unwin, 1994 ▸; Walker *et al.*, 1995 ▸; White *et al.*, 1998 ▸; Unwin & Fujiyoshi, 2012 ▸). This simple concept is applicable to reactions where the sprayed component is small and rapidly diffusing. Examples include the dynamic activation of myosin by ATP on actin filaments, and relaxation of the acetylcholine (ACh) receptor in response to ACh (Table 1 ▸). This approach could be applicable to many interactions between biological macromolecules and small molecules, such as drugs, signalling molecules and buffer components, or even the pH or temperature of the buffer could be varied. It is less suitable for questions that involve the interaction with other biological macromolecules, as the degree to which the two fluids mix is poorly controllable and likely to be inefficient (Castrejón-Pita *et al.*, 2013 ▸).

The above approach has recently been used for a trEM adaptation of the Spotiton system (Dandey *et al.*, 2018 ▸). In brief, the Spotiton approach relies on self-wicking grids as an alternative to blotting paper for thinning out the liquid. Copper nanowires on the grid surface act as a wicking surface to absorb excess liquid. In a natural extension into time-resolved studies, two streams of droplets delivered by independent piezoelectric dispensers were consecutively applied onto the support grid (Fig. 4*c*). This method allowed visual­izations of structural changes in the ion channel MthK as well as well as several binding events in ribosomes, RNAP and dynamin (Dandey *et al.*, 2020 ▸).

More defined rapid mixing methods for trEM require microfluidic manipulation of the reactants prior to deposition on a support grid (Figs. 2 ▸ *c* and 2 ▸ *d*). The most straightforward and widely used method adapts a T-mixer geometry, which merges two fluid streams in a tube, resulting in one major liquid–liquid interface across which diffusive mixing occurs (Fig. 2 ▸ *c*; White *et al.*, 1998 ▸, 2003 ▸; Kontziampasis *et al.*, 2019 ▸). The mixing efficiency of T-mixers is often lower than desirable in low Reynolds number flows as beyond the initial inter­section mixing proceeds by diffusion only (Fig. 2 ▸ *c*), and thus more elaborate microfluidic mixing geometries are required to ensure that mixing is both efficient and rapid. One very successful method (Lu *et al.*, 2009 ▸; Shaikh *et al.*, 2014 ▸) is to use a T-type mixer followed by four butterfly mixing geometries, which essentially split and re-merge the fluid stream multiple times, generating convective mixing within the channel. Another method employs a mixing geometry which uses a series of three-dimensional turns within the channel, optimized to generate very rapid mixing while keeping manufacturing simple by using PDMS as a material (Kim *et al.*, 2016 ▸; Mäeots *et al.*, 2020 ▸). The latter two approaches have been shown to enable 96% and >90% mixing efficiency, respectively, through a fluorescent experimental assay (Fig. 2 ▸ *d*; Mäeots *et al.*, 2020 ▸; Lu *et al.*, 2010 ▸). In the particular case of on-grid mixing, quantification is not straightforward; however, a discussion of this topic can be found in Klebl *et al.* (2021 ▸). Using rapid mixing methods ensures that as many of the reactant molecules as possible begin their interactions at the desired zero time point. Microfluidic mixing is very generalizable to different types of reactants and achieves high degrees of mixing, with the potential downside that these methods use flow rates of around 6 µl s^−1^ per channel to achieve highly efficient mixing, which increases sample consumption.

While most time-resolved interactions require the mixing of two components, there are noteworthy special cases that do not. Chang & Reese (1990 ▸) showed that an electrical pulse can be delivered before freezing to induce a structural change in cell membranes. This greatly simplifies the problem as the entire sample can be affected simultaneously and mixing is not required. A further subset of biological questions can be answered using photolabile caged ligands to trigger reaction initiation (Shaikh *et al.*, 2009 ▸). Adopting a well known method from time-resolved X-ray crystallography, the protein and chemically modified ligand can be premixed, only starting the reaction with a flash of light and stopping it by vitrification on timescales below 100 ms (Bernardinelli *et al.*, 2005 ▸; Yoder *et al.*, 2020 ▸). A further reported use of a laser light source was to temporarily devitrify the sample so that it can undergo conformational change, potentially to be combined with releasing a caged ligand (Voss *et al.*, 2021 ▸). The compatibility of this innovative approach with retaining high-resolution structural information remains to be tested, but it holds the promise of visualizing structural transitions on microsecond timescales.

## 3. Controlling incubation time

After the reaction has been initiated, a precise incubation time is required to freeze the reaction at time points where the species of interest are most abundant. For reactions which take more than a few seconds to complete this step is less critical, as incubation can be performed in a test tube followed by standard cryo-EM sample preparation (Miller *et al.*, 2019 ▸; Mandelkow *et al.*, 1991 ▸; Siegel *et al.*, 1989 ▸; Heymann *et al.*, 2004 ▸). Alternatively, as described above, samples can be mixed and incubated on the grid after blotting (Fig. 1 ▸ *a*). Although attractive in its simplicity, the concern with this type of incubation is that the reactants are spread into a thin film, potentially interacting with non-native air–water or air–carbon interfaces which can impede free diffusion or lead to structural artefacts (Glaeser, 2018 ▸; D’Imprima *et al.*, 2019 ▸; Glaeser *et al.*, 2016 ▸).

When studying more rapid processes such as the activation of transmembrane channels or ribosome association, which occur within the millisecond range (Fig. 1 ▸), automation needs to be engineered. In the simplest case, the reaction incubation time is limited by the dead time of the device, meaning that the reaction time is determined by how rapidly the steps from mixing the samples to freezing are performed (Trachtenberg, 1998 ▸; Lu *et al.*, 2009 ▸; Feng *et al.*, 2017 ▸; Kontziampasis *et al.*, 2019 ▸; Dandey *et al.*, 2020 ▸). Due to the mechanical nature of plunging a grid into ethane, current devices tend to require upwards of 10 ms to freeze the grid, as increasing the speed of the machine much further would become prohibitive from a cost and safety perspective (Kasas *et al.*, 2003 ▸). Although faster devices could be imagined, it is not clear that this is the major limiting factor at the moment.

**Figure 3:**
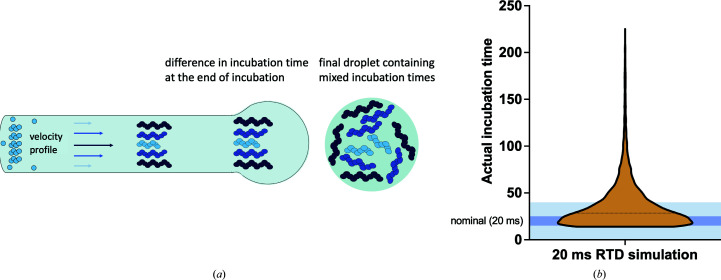
Effects of laminar flow on the sample during incubation. (*a*) Different layers of liquid move at different velocities in the channel, generating a distribution of incubation times. This is represented as different lengths of filament growth (Mäeots *et al.*, 2020 ▸). When a droplet is generated at the end of the channel it contains a mixture of all species, in accordance with their prevalence. (*b*) Histogram of the residence-time distribution (RTD) due to laminar flow. Dark blue is 15–25 ms and contains 33% of the total data. Light blue is 0–40 ms and contains 70% of the total data. For further details, see Mäeots *et al.* (2020 ▸).

On the other hand, if the reaction needs to be incubated for a precisely defined amount of time, within millisecond bounds, it is most commonly approached by increasing the distance between the mixing region and the point where the sample is sprayed in a so-called ‘delay line’ (White *et al.*, 2003 ▸; Frenz *et al.*, 2009 ▸). Assuming a well known relationship between time and distance travelled at constant average velocity, incubation times can be precisely engineered through micromanufacturing of the required channel lengths. In the vast majority of practical cases the Reynolds numbers characterizing the microfluidic flows are very small, *i.e.* they are in the laminar flow regime. In this regime, mixing can be difficult due to lack of flow or eddies perpendicular to the flow direction. This further manifests as different fluid velocities across the channel (Fig. 3 ▸ *a*). This will prevent the even incubation of the sample within a microfluidic device as reactants closer to the centre of the channel spend much less time interacting in the device than those closer to the edges (Fu *et al.*, 2019 ▸; Mäeots *et al.*, 2020 ▸; Klebl *et al.*, 2021 ▸). Thus, the average incubation time for a fixed channel length and volumetric flow rate is not a good description of the actual residence-time distribution of the reactants, which is parameterized by a nonsymmetric Poisson distribution (Fig. 3 ▸ *b*). Moreover, this effect becomes more severe as the length of the incubation channel increases, meaning that incubation times of over a second in length are better approached by other methods such as incubation in the absence of flow. Despite these idiosyncrasies, laminar flow is a linear process and its effects can be simulated reliably. An important future research direction is the use of such simulation alongside experimental data for precise quantification and the development of methods for correcting the introduced errors to observable time resolution.

## 4. Achieving sufficient time-resolved cryo-EM sample quality within millisecond timescales

The fundamental challenge for cryo-EM sample preparation is the requirement for a thin layer of frozen hydrated sample (Dubochet *et al.*, 1988 ▸). In standard methods the sample applied to the support grid is thinned down to less than 100 nm by blotting. This process commonly utilizes blotting paper and takes about a second or longer to complete. Thus, method developments aiming at trEM on faster timescales necessitate alternative, more rapid, methods of generating thin ice.

**Figure 4:**
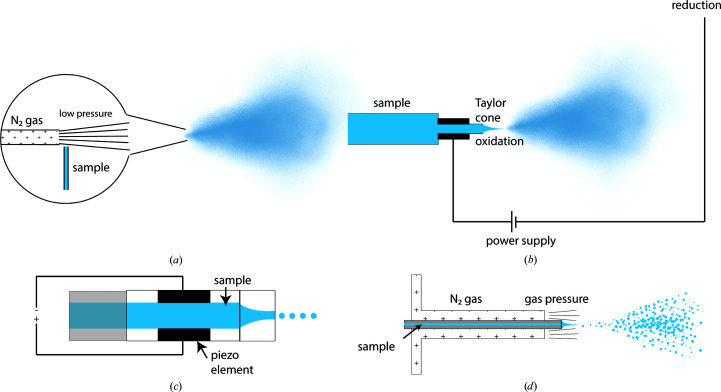
Sample-dispensing methods. (*a*) Atomizer spray, using Bernoulli’s principle to generate very small sample droplets. (*b*) Electrospray nozzle, which uses ionization of the sample to generate fine mists of droplets at the output channel without the use of gas pressure. (*c*) Inkjet nozzle, using a piezoelectric element to generate acoustic vibrations in the sample at a fixed frequency, leading to uniform droplet formation. (*d*) Gas-assisted spray, where sample is continuously pumped through the channel and broken up by gas pressure at the exit, which generates small droplets as well as accelerating the spray towards the grid. Gas-dynamic virtual nozzles (GDVNs) are conceptually similar and enable greater aerosol control through more sophisticated manufacturing (Klebl, Monteiro *et al.*, 2020 ▸).

In the simplest cases, activating pulses of light releasing a caged compound are applied to the already blotted sample, thus triggering and incubating the reaction while plunging (Chang & Reese, 1990 ▸; Yoder *et al.*, 2020 ▸; Voss *et al.*, 2021 ▸). In this case depositing a thin layer of sample on the grid is not time-sensitive. In order to provide a general method for rapid sample preparation, a different technology is required. This is achieved by applying smaller volumes to the grid (usually in the form of an aerosol), thereby reducing the amount of thinning required, which saves the time needed to blot. Alternatively, this can also be achieved by blotting more rapidly in the case of self-wicking grids, as discussed above. The very first reported trEM studies are a specialized case of aerosol delivery combined with on-grid mixing (Fig. 2 ▸ *b*). The technical innovation was the use of an atomizer, blowing one reactant through a channel containing a glass sphere, which breaks the incoming jet into droplets of roughly 1 µm in size, onto the grid containing the second reactant in a thin film (Berriman & Unwin, 1994 ▸; Walker *et al.*, 1995 ▸). This spraying device differs from most recently reported solutions, since the grid is already pre-wetted with the first reactant and the reaction would only start once the droplets hit the grid (Fig. 4 ▸ *a*). While this was an effective means of delivering sample rapidly onto the grid, it bypassed two major problems with spraying methods. The first is that in this on-grid mixing the spray itself did not need to consider residence time within the nozzle, and the second is that by pre-wetting the grid with the first sample, the wetting properties of the droplets hitting the grid could effectively be ignored. This limits the approach to aerosolized reactants that can diffuse rapidly, effectively preventing this being used for protein–protein interactions.

A general solution which can time-resolve the reaction between any species will require premixing the samples using microfluidics. For samples mixed in microfluidic channels, the following problem becomes limiting: how to convert the sample from a microfluidic channel into a thin film on a cryo-EM support grid within the necessary timescale and without including any additional loss of time resolution? The first method which uses premixed samples was described by White *et al.* (1998 ▸), which uses a different atomizer design utilizing voltage-assisted spraying as well as a T-mixer to generate a mixture of two reactants (Fig. 4 ▸ *b*). This concept could be adapted to produce thin ice using microfluidic mixing; however, the sample was first sprayed onto the grid in excess, followed by blotting. While effective, this set a lower bound of ∼1 s on the achievable time resolution and therefore could be improved upon further, as described in White *et al.* (2003 ▸). Voltage-assisted spraying has been further developed by Kontziampasis *et al.* (2019 ▸), where the approach was adapted to produce thin ice over dry grids, removing the need for additional blotting.

The first device to demonstrate both rapid microfluidic mixing and spraying with no prior or further manipulation was reported by Lu *et al.* (2009 ▸). This device used gas-assisted spraying rather than voltage to generate droplets and found that larger droplets of around 10 µm in size were actually preferable for spreading on dry, glow-discharged grids (Fig. 4 ▸ *d*). However, the number of areas suitable for data collection per grid was still much lower than for grids produced using slower, blotting methods. This was then further developed upon when the 2D nozzle design was replaced with a 3D annual gas-assisted micronozzle, which produced droplets in a much narrower range of sizes, generating more usable ice per grid (Lu *et al.*, 2014 ▸). This same idea was then exchanged for an internal-mixing round-jet microsprayer and represented a further increase in collectable area per grid, with average thicknesses below 100 nm (Feng *et al.*, 2017 ▸). Collectively, these innovations have enabled some of the most sophisticated and reliable trEM studies to date, enabling new biological insights into translation on millisecond timescales (Table 1 ▸).

Another variant of gas-assisted spraying is presented by Mäeots *et al.* (2020 ▸), in which the liquid jet is broken up by a concentric ring of pressurized gas at the exit of the device. By varying both the size of the concentric gas ring and the gas pressure, we were able to optimize the resulting aerosol to reach a satisfactory number of areas per grid so that a data set could be collected from a single grid, although the average ice thickness was over 100 nm. This was then successfully applied to follow nucleation and growth of RecA filaments over a time course with millisecond timescales (Table 1 ▸).

Overall, rapid progress has been made in this area over the last few years, with at least three techniques able to deliver near-atomic resolution cryo-EM data in less than 30 ms. However, future improvements to rapid sample manipulation will likely bring equal or even greater control over sample application and thickness compared with conventional cryo-EM sample preparation.

## 5. Benefits of rapid sample preparation

In addition to trapping transient intermediates, rapid sample preparation has been found to have other benefits. By preparing the sample in a few milliseconds, we and others found that the sample did not strongly concentrate at the air–water interfaces (Mäeots *et al.*, 2020 ▸; Dandey *et al.*, 2020 ▸), although Klebl, Gravett *et al.* (2020 ▸) provide conflicting observations. This is a well known effect that can hinder many sample-preparation methods (D’Imprima *et al.*, 2019 ▸; Klebl, Gravett *et al.*, 2020 ▸; Glaeser, 2018 ▸). A protein contacting air on the surface of the thin film can exhibit preferential orientations and/or partially denature, leading to loss of reconstruction resolution and quality. These interactions are very rapid and can occur thousands of times per second, although not all interaction events lead to denaturation. However, over the course of time the effect becomes substantial. A less beneficial consequence of time-resolved sample preparation is the lower sample concentration observed compared with conventional blotting methods (Klebl, Gravett *et al.*, 2020 ▸). Both of the above observations are likely to result from the same underlying process. When performing standard blotting the thin film is stabilized for seconds, presenting many opportunities for the sample to make contacts with the air–water interfaces (Glaeser, 2018 ▸). As the excess liquid is removed during blotting, a concentrating effect ensues due to sample remaining stuck at these interfaces, alongside partial or total denaturation of some portion of this sample. Not all biomolecules clustering at the air–water interface denature, but the effect can additionally restrict the available viewing directions of the sample. This common issue, which is colloquially referred to as orientational bias, is a major challenge for present single-particle data-analysis methods and can limit resolution and generate artefacts. Limiting this bias is a key factor in producing high-resolution reconstructions with current reconstruction algorithms (Naydenova & Russo, 2017 ▸). In contrast, during time-resolved sample preparation both effects are reduced simultaneously, leading to the observation of both less concentrated and less damaged samples. These benefits were also observed using rapid grid preparation with a Chameleon system, the commercial version of Spotiton (Levitz *et al.*, 2022 ▸). These benefits, along with the automated nature of all time-resolved sample-preparation techniques, could prove very attractive to structural biologists in the future.

## 6. Remaining challenges and future directions

Time-resolved cryo-EM has seen rapid development from a growing community in the last few years. The technique is maturing and its potential is widely accepted. However, adoption of the method will depend on further improvements to areas such as reducing sample consumption and improving ice thickness and reproducibility, as well as developing new useful features such as optical quality control of the process as well as *in situ* spectroscopic analysis of the biochemical reactions. Another key limitation is the lack of access to affordable devices for time-resolved sample preparation outside the developing laboratories, which will require engineering and entrepreneurial efforts to resolve.

Achieving parity with standard sample-preparation methods in terms of sample quality and tuneable parameters to fit each sample remains an urgent priority. Current time-resolved experimental setups are not easy to operate by nonspecialists and adjusting settings requires expert validation. Moreover, sample consumption is a major burden. These and related issues can be improved through the budget-conscious development of reliable and simple-to-use mass-manufacturable devices that combine robust mechanical assemblies and in-line quality-control sensors with intuitive software control. As the method of trEM will ultimately only be as useful as the biological insights which can be gained from it, the importance of carefully aligning the efforts of structural biologists and engineers towards these goals in both academic and industrial settings cannot be overemphasized.

A hitherto unexplored direction is method developments that ensure technical parity between measured kinetic parameters and the trEM experiment. An important input parameter at the onset of a trEM experiment is the reaction incubation time. A well known challenge among biophysicists is the difficulty of exactly reproducing reported kinetic measurements across experimental setups due to the large effects of many possible small variations and the high precision of the readouts. One possible solution to this is to enable *in situ* kinetic measurements through engineering integration. For microfluidic-based trEM sample-preparation setups this could be achieved by introducing a valve system which stops the flow rapidly after the initial mixing of the reagents. Furthermore, an optical system could be integrated which would enable monitoring the progression of the reaction as a function of, for instance, a fluorescent signal such as a FRET reporter pair. This approach would increase the scientific accuracy of trEM studies and reduce the need to sample the temporal space of the studied reaction by cryo-EM and single-particle analysis, which would be both laborious and costly.

In the longer term, the availability of time information would need to be integrated into the data-processing pipeline. An essential prerequisite for achieving this is the accurate mapping of observed conformations to the time coordinates of the reaction. Due to the laminar flow effects illustrated in Fig. 3 ▸, obtaining such precise timing is presently not straightforward. Instead, it is presently necessary to produce computational simulations of the predicted outcome for this purpose. Moreover, the possibilities of experimental and systematic errors such as imperfect mixing or loss of sample inside the microfluidic device need to be assessed in dedicated experiments (Mäeots *et al.*, 2020 ▸). A very attractive future direction to integrate these two orthogonal control experiments is to develop kinetic model systems with well characterized properties in order to examine the behaviour of the system as a whole: a concept related to measuring the linear response function of an engineered system. Algorithmically inverting this experimentally determined response through deconvolution will be key to enabling reliable and routine mapping of conformational states to reaction coordinates. In parallel, computational approaches such as a manifold embedding or latent embedding are being actively explored to enable a comprehensive description of all observed computational states in a given cryo-EM data set (Frank & Ourmazd, 2016 ▸; Zhong *et al.*, 2019 ▸; Wu *et al.*, 2020 ▸; Punjani & Fleet, 2021 ▸). In the future, a synthesis of these approaches will attempt to map all the individual cryo-EM observations of a particle to a conformational space that most accurately describes the structural transitions. At the moment these approaches do not incorporate time information; however, future developments could integrate this.

The combination of precise methodology and accurate kinetic measurements has offered inspiring novel insights into macromolecular dynamics (Kaledhonkar *et al.*, 2019 ▸). With continued efforts towards improving time-resolved structural biology methods and enabling their wide adoption, it will eventually become routine to study biological macromolecules as dynamic nodes in complex networks, which react and adapt through conformational changes to various biological cues such as tension, metabolites, binding partners or covalent modifications. A more complete picture of the conformational space of proteins and its relationship to their function is bound to greatly advance basic and applied science. The potential for harnessing such information to open new avenues of drug discovery or the ability to comprehend the basic principles of life are great sources of motivation for future efforts.

## Figures and Tables

**Table 1:** Overview of reported time-resolved cryo-EM studies

trEM system	Biological system	Time resolution	Reference
Manual mixing	Phospholipid membrane fusion	3–6 s	Siegel *et al.* (1989 ▸)
Electric pulse delivery	Membrane electroporation	3 ms–10 s	Chang & Reese (1990 ▸)
Light pulse delivery	Bacteriorhodopsin	10–20 ms	Subramaniam *et al.* (1993 ▸)
Light pulse delivery to release caged compound	Acid-sensing ion channel	70 ms	Yoder *et al.* (2020 ▸)
Light pulse delivery	GroEL chaperone disassembly	10–50 µs	Voss *et al.* (2021 ▸)
On-grid mixing	Vesicle osmosis	1–100 ms	Berriman & Unwin (1994 ▸)
On-grid mixing	Myosin	10 ms	Walker *et al.* (1995 ▸)
On-grid mixing	Acetylcholine receptor	10 ms	Unwin (1995 ▸), Unwin & Fujiyoshi (2012 ▸)
On grid mixing	Vesicle formation	7–70 ms	Adams *et al.* (2009 ▸)
On-grid mixing	MthK activation	90–150 ms	Dandey *et al.* (2020 ▸)
Microfluidic mixing and spraying	Ribosome association	9.4 ms	Barnard *et al.* (2009 ▸)
Microfluidic mixing and spraying	Ribosome association	9.4–34 ms	Shaikh *et al.* (2014 ▸)
Microfluidic mixing and spraying	Ribosome association	60–140 ms	Chen *et al.* (2015 ▸)
Microfluidic mixing and spraying	Bacterial translation	20–600 ms	Kaledhonkar *et al.* (2019 ▸)
Microfluidic mixing and spraying	Ribosome association	24–60 ms	Fu *et al.* (2019 ▸)
Microfluidic mixing and spraying	Myosin	15 ms	Kontziampasis *et al.* (2019 ▸)
Microfluidic mixing and spraying	RecA recombinase filament growth	30–1360 ms	Mäeots *et al.* (2020 ▸)
